# Effects of Growth Hormone Administration on Muscle Strength in Men over 50 Years Old

**DOI:** 10.1155/2013/942030

**Published:** 2013-12-08

**Authors:** A. B. W. Tavares, E. Micmacher, S. Biesek, R. Assumpção, R. Redorat, U. Veloso, M. Vaisman, P. T. V. Farinatti, F. Conceição

**Affiliations:** ^1^Endocrine Service, Clementino Fraga Filho University Hospital, Federal University of Rio de Janeiro, Rua Barão de Lucena, 135/202 Botafogo, 22260-020 Rio de Janeiro, RJ, Brazil; ^2^Physical Activity and Health Promotion Laboratory (LABSAU), Rio de Janeiro State University, Rio de Janeiro, RJ, Brazil; ^3^Salgado de Oliveira University, Rio de Janeiro, RJ, Brazil

## Abstract

Growth hormone (GH) use has been speculated to improve physical capacity in subjects without GH deficiency (GHD) through stimulation of collagen synthesis in the tendon and skeletal muscle, which leads to better exercise training and increased muscle strength. In this context, the use of GH in healthy elderly should be an option for increasing muscle strength. Our aim was to evaluate the effect of GH therapy on muscle strength in healthy men over 50 years old. Fourteen healthy men aged 50–70 years were evaluated at baseline for body composition and muscle strength (evaluated by leg press and bench press exercises, which focus primarily on quadriceps—lower body part and pectoralis major—upper body part—muscles, resp.). Subjects were randomised into 2 groups: GH therapy (7 subjects) and placebo (7 subjects) and reevaluated after 6 months of therapy. Thirteen subjects completed the study (6 subjects in the placebo group and 7 subjects in the GH group). Subjects of both groups were not different at baseline. After 6 months of therapy, muscle strength in the bench press responsive muscles did not increase in both groups and showed a statistically significant increase in the leg press responsive muscles in the GH group. Our study demonstrated an increase in muscle strength in the lower body part after GH therapy in healthy men. This finding must be considered and tested in frail older populations, whose physical incapacity is primarily caused by proximal muscle weakness. The trial was registered with NCT01853566.

## 1. Introduction

The ageing process is accompanied by an increased prevalence of the signs and symptoms of physical fragility, including sarcopenia, a decrease in exercise tolerance, osteopenia, an increase in visceral adiposity and a worsening of quality of life. Adults with growth hormone deficiency (GHD) exhibit similar clinical signs, but it is possible to treat them with GH to ameliorate these signs and symptoms [1].

The GH secretion declines gradually with age, whereby studies demonstrate a progressive reduction of 14% secretion per decade of life beginning in the second decade [2, 3]. These findings suggest a possible association between GHD and the ageing process [4, 5]. The association between the physiological alterations of the ageing process and the reduction of GH secretion is called somatopause.

GH replacement is well known to improve body composition, leading to a decrease in total body fat and an increase in lean body mass [1]. However, GH replacement has only shown an effect on muscle strength in GH-deficient adults subjected to long-term GH therapy [6]. Few studies have evaluated the effect of GH replacement on muscle strength in elderly people engaged in a program of exercise training [2, 7–10]. Specific training that aims to increase muscle strength can potentiate the anabolic effect of GH, which leads to a greater impact on body composition in comparison to GH replacement alone [11, 12]. Adults with GHD demonstrated normalisation of muscle strength after long-term GH replacement [6]. These subjects showed increased exercise capacity with improvements in oxygen uptake and ventilatory threshold after GH therapy. This improvement was most likely due to some combination of increased muscle strength, improved body composition, and improved thermoregulation [13]. GH has been speculated to improve physical capacity in subjects without GHD through stimulation of collagen synthesis in the tendon and skeletal muscle [14], which leads to better exercise training and increased muscle strength. Administration of supraphysiological doses of GH to athletes increases fatty acid availability, reduces oxidative protein loss, particularly during exercise, and increases lean body mass [13].

Our purpose was to evaluate the effect of GH therapy on muscle strength in healthy, nonsedentary men over 50 years old.

## 2. Subjects and Methods

### 2.1. Subjects

Healthy and nonsedentary men ageing 50–70 years were recruited by newspaper advertisements with information that patients were needed for a study of hormonal evaluation. Exclusion criteria were pituitary disease, GH use in the last 12 months, severe acute disease, hepatic and/or renal chronic disease, uncontrolled systemic arterial hypertension, diabetes mellitus, psychiatric disorders, history of cancer, nontreated hypogonadism, and the presence of any other disease that could interfere with the somatotrophic axis.

All patients practiced some kind of exercise (minimum of three times per week, aerobic and resistance exercise) to be eligible to be included in the study.

Twenty-nine subjects were screened for a project about somatotrophic profile in healthy men over 50 years old (which includes this paper) and have resulted in other published studies [15, 16]. After that, fourteen from the 29 initial patients agreed to participate in this part of the project that included the evaluation of muscle strength in GH x placebo replacement; those subjects who gave up had personal problems, such as work compromises and address change. All patients signed the informed written consent approved by the Ethics Committee of the Clementino Fraga Filho Hospital/UFRJ.

### 2.2. Methods

At baseline, subjects were submitted to evaluation of GH secretion, testosterone level, body composition, and muscle strength. Absence of pituitary disease and normal testosterone levels were necessary for the inclusion in the study.

GH secretion was evaluated through insulin tolerance test (ITT) or glucagon stimulation test (GST) and IGF-I levels. The ITT was performed by intravenous injection of regular insulin at a dose of 0.1–0.15 IU/Kg. Blood samples were drawn every 30 minutes from baseline until 120 minutes for the determination of glucose and GH levels. All patients had hypoglycemia (glucose nadir lower than 40 mg/dL) during the test. The GST was performed by intramuscular injection of 1 mg of glucagon. Blood samples were drawn every 30 minutes from baseline to 180 minutes. Severe GHD was considered when GH peak was lower than 3 *μ*g/dL, and it was an exclusion criterion.

GH analyses were performed by an immunometric assay (Immulite). The minimal detection limit was 0.01 *μ*g/L and the intra-assay coefficient of variation (CV) was 5.3% at 1.7 *μ*g/L. The interassay CV was 5.7% at 3.0 *μ*g/L. Serum IGF-I concentrations were determined by an immunoradiometric assay (Kit DSL-5600), with acid-ethanol extraction. The minimal detection limit was 0.80 ng/mL and the intra-assay was 3.4% at 9.38 ng/mL and 3.7% at 255.8 ng/mL.

All subjects had GH peak at GST higher than 3 *μ*g/L and had IGF-I levels normal according to their age; that is, none of them had severe GHD (Table 1). Testosterone levels were normal in all participants of the study.

Subjects were then randomised according to their arrival to the medical evaluation: one patient to each group (GH and placebo groups), sequentially.

The GH group received an initial dose of 0.5 UI/day (0.2 mg/day), with readjustments to 1.0 UI/day (0.4 mg/day) and 1.5 UI/day (0.6 mg/day) after 1 and 2 months of treatment, respectively. The last GH dose was maintained until the end of the study. The second group received placebo using the same scheme as the GH group. These GH dosages were based on the dose used for adults of the same age with GHD at the time of this research. The GH was supplied by Pfizer Laboratory (Genotropin). Subjects were reevaluated after 6 months of GH therapy or placebo according to the parameters above. Pfizer Laboratory also provided the placebo.

During treatment, every month the patients should take their used GH flask to the medical consult in order to realize a counting flask for control of the correct use of GH.

#### 2.2.1. Body Composition

Body mass index was calculated using Quetelet's formula (weight/height^2^) and the percentage of body fat was calculated using the adipometer on seven cutaneous folds (tricipital, subscapular, suprailiac, pectoral, axillary, abdominal, and thigh) according to the Pollock and Jackson protocol [17]. Abdominal circumference was measured using a nonelastic tape at the greatest diameter between the last ribs and the iliac crests. The same person examined all subjects.

#### 2.2.2. Muscle Strength

Muscle strength was evaluated for the maximum strength based on the concept of maximum repetition (1 MR) (i.e., the maximum load that can be performed using the correct technique for an exercise) [18].

The exercises evaluated were bench press and leg press using a Righetto machine (São Paulo, Brazil), which are dynamic exercises. All patients were evaluated through both exercises. The bench press focuses on the strengthening of the pectoralis major muscle as well as other muscles including anterior deltoids, serratus anterior, coracobrachialis, scapulae fixers, trapezzi, and the triceps. The bench press was performed according to the following technique: (a) initial position: dorsal decubitus, elbows in extension with hands sustaining the barbell, knees and hips semiflexed, and feet on the equipment base; (b) exercise development: elbows flexion, to form a 90 degree angle with the arm and forearm, followed by complete extension of the elbows and horizontal flexion of the shoulders, returning to the initial position. The leg press works the following muscles groups: quadriceps, hamstring, gluteus maximus, and calves (partially). The leg press was performed according to the following technique: (a) initial position: hips and knees flexed at a 90 degree angle, feet on the platform, and inferior members slightly separated and lined up within the limits of the iliac crests; (b) exercise development: complete extension of the knees then returning to the initial position.

Before the use of the equipment, subjects could familiarise themselves with the exercises and the equipment without the load. To determine the load associated with 10 MR, each subject initially performed 10 repetitions of each exercise with a submaximum load, which was considered a load that was possible to mobilise. Then, the loads were progressively increased and, with a maximum of 3 trials, the load of 10 MR was reached. If the 10 MR load was not reached after 3 attempts, the subject was asked to come to the laboratory for another opportunity. To reduce the error margin, the following strategies were adopted: (a) standardised instructions were given before the test to make the subjects aware of the data collection routine; (b) subjects were instructed on the execution technique of the exercises; (c) the examiner was attentive to the correct position of the patients' joints at the time of measurement to avoid small variations in joint positioning that could put action on other muscles and lead to erroneous interpretations of the obtained scores; and (d) verbal stimuli were given during the exercise to maintain a high level of motivation. During the 10 MR test, a 2–5 minute interval was provided between the attempts of each exercise. After the load was obtained for a determined exercise, intervals not less than 10 minutes were given before moving on to the next exercise.

#### 2.2.3. Statistical Analysis

Comparisons between the groups and timepoints were performed using two-way ANOVA for repetitive measures, followed by Fischer *post-hoc* evaluation if necessary. The differences between the baseline and the last measure (delta) were compared by Student's *t*-test for independent variables. A *P* value <0.05 was considered statistically significant.

## 3. Results

Thirteen subjects completed the study (6 subjects in the placebo group and 7 subjects in the GH group). One subject in the placebo group dropped out of the study because of the development of arthralgia. No patient in the GH group had any potential side effects. Subjects of both groups were similar in age, weight, height, and BMI at baseline; they were overweight according to BMI (between 25–30 Kg/m^2^), except one subject who was obese (BMI 38.5 Kg/m^2^)—Table 1. All subjects of both groups were nonsedentary; that is, they performed planned physical activity for more than 150 minutes per week.

After 6 months of therapy, the evaluation of the GH and placebo groups did not show evidence of significant differences in weight, BMI, waist, and the sum of the cutaneous folds (triceps, biceps, subscapular, and iliac folds). Furthermore, there were no differences on the delta (6 months—baseline) for these parameters between the groups (Table 2).

Muscle strength in the upper body part, as evaluated by supine horizontal rising, did not increase after 6 months in both groups. Muscle strength in the lower body part, as measured by the leg press, showed a statistically significant increase in the GH group when compared to placebo (Table 2).

## 4. Discussion

The effects of ageing cause undesirable changes in body composition, a reduction of bone mineral density and muscle strength (with greater falling risk), and worsening exercise capacity, which is associated with a decrease in GH and IGF-I levels. However, GH replacement to minimise the adverse effects of ageing must be based on cost-benefit and risk-benefit analysis. Furthermore, randomised studies demonstrated that the effectiveness of GH replacement is modest, either on its own or in combination with sex steroids or exercises [2, 4, 7].

Our study evaluated muscle strength in men over 50 years of age after 6 months of GH therapy, and we observed a statistically significant increase in leg press responsive muscles (quadriceps being the mainly muscle evaluated) when compared to placebo. There was no evidence of alterations in body composition in either group (GH therapy or placebo). The GH group had a BMI and body fat percentage (represented by the sum of the cutaneous folds) slightly greater than the placebo group but without statistical significance. These parameters did not change after 6 months of therapy. Therefore, our results differed from those found in the literature, in which an improvement in body composition and no difference on muscle strength during GH replacement are described [7, 9].

The effects of GH therapy on body composition (increased lean body mass and decreased fat mass) are very well reported [1, 19–24], but the effects on muscle strength are unclear, which was one of the objectives of our study. It has already been reported that recombinant human GH administered for 6 months to healthy older men decreases the percentage of body fat and increases lean body mass and IGF-I levels to values observed in young adult males [24]. A recent study using a low-dose sustained-release recombinant human GH administered during 26 weeks to subjects with the so-called somatopause showed improvements in body composition and quality of life [25].

Many studies in GHD adults demonstrated the normalisation of muscle strength after 10 years of GH replacement [6, 26, 27] but did not show an increase in muscle strength with the use of GH for short periods of time. However, a study of 18 elderly men submitted to progressive weight training for 14 weeks and then randomised to receive either GH or placebo during a further 10 weeks of strength training showed that GH had no effect on muscle strength at any time, but lean body mass increased and fat mass decreased in the GH group [7]. Frail older people subjected to GH treatment with or without a structured resistance exercise program had a significantly increase in muscle strength in both GH/exercise as well as the exercise alone groups, but there was a significant increase in the proportion of type 2 fibers at the end of the study in the combined GH/exercise treated subjects [28].

There are few studies in the elderly that combine GH replacement and specific training for muscle strength [7, 8]. This type of training could potentiate the anabolic effects of GH, which would cause a greater impact on body composition than GH replacement alone as well as greater impact on muscle strength. GH has been speculated to improve physical capacity in subjects without GHD through its stimulation of collagen synthesis in the tendon and skeletal muscle [14]. Furthermore, GH appears to be more important for the reinforcement of the tissue matrix than for muscle cell hypertrophy [29]. Thus, the GH use could attenuate or prevent muscle fibre injuries, causing a lower frequency of training disruption and leading to greater training in athletes or nonathletes with the use of GH, which leads to a muscle fibre hypertrophy and consequently an increase in strength. This could explain the increase in leg press responsive muscles in the GH group of the present study. Although no participants of our study were athletes or performed supervised physical training before and during the GH x placebo use, none of the subjects were considered sedentary and all practiced some type of exercise (minimum of three times per week—150 minutes per week, aerobic and resistance exercises). We can postulate that this group, even without supervised training, had fewer injuries and consequently performed more exercises to increase muscle strength. Moreover, the majority of subjects realized walk and running as exercise, which prioritizes the lower body part muscles.

Another point that corroborates the gain of muscle strength in this group is the documented evidence that adults with GHD have lower fatigue sensation when subjected to GH replacement, which increases the adherence to exercise and leads to cardiopulmonary benefits [30]. Although there are conflicting results regarding adults without GHD, it could be another fact that increased the disposition to exercise and consequently to an increase in lower body part strength in subjects in the GH group.

The median age of the subjects in our study was lower than the studies cited above. Although our objective did not include evaluation of the gonadic axis, subjects were required to have normal serum levels of testosterone to be included in the study. Thus, we can attribute the observed amelioration of strength in leg press responsive muscles to the GH therapy, independent of gonadic function. Another point is that the majority of the studies were performed for periods less than 6 months, and this could be an explanation for the different results obtained in our study. Another consideration is the GH dosage used in our study; we used a fixed dosage with increases in the first and second months, independent of the patient's weight. The final GH dosage (0.6 mg/day) was the median dosage used for adults (men with an average age of 50 years) with GHD at the time that our study began [19]. However, at the present data, GH dosages are lower in GHD adult replacement (initial dose of 0.1–0.3 mg/day) [1]. We cannot discard a pharmacologic effect of this higher GH dosage on muscle strength.

Although anthropometry is an important method of nutritional evaluation in subjects over 50 years of age, one limitation of our study is that we did not use a more precise method of body composition evaluation. Fat tissue redistribution is more prominent in women, and reduction in body water content is more common in men [31]. In addition, the lack of references for anthropometric measures in the Brazilian population [32] can reduce the precision of such evaluations. The use of dual energy X-ray absorptiometry (DEXA) would improve evaluation of modifications in body composition, mainly lean mass. Even still, the results could still be vulnerable to error because DEXA is not able to estimate body water accumulation caused by GH therapy. We used anthropometric indicators for low-cost and easy application. The BMI was demonstrated to be well correlated with anthropometric indicators of nonvisceral fat (tricipital and subscapular folds) and abdominal fat (abdominal circumference), in addition to being in direct relation with total body fat mass [33, 34]. The fact that we did not find a change in weight in the study group despite an improvement of strength in leg press responsive muscles might suggest that there was an increase in muscle mass and a decrease in fat mass.

Our study differs from the literature in the following parameters: (1) age (our population was younger); (2) exercise training (subjects in our study were not considered sedentary and did some type of exercise but not a specific training); (3) GH dose (similar to the dose used for GHD adult men at the time of the study); and (4) longer duration of GH use (6 months).

Our study demonstrated an increase in leg press responsive muscles in men over 50 years old after GH therapy, which is important for reducing falls and fractures in aged populations. This finding must be considered and tested in frail older populations, whose physical incapacity is caused primarily by proximal muscle weakness. Considering that ageing is related to loss of strength in lower body part and a worsening quality of life, our results can be taken into account for future studies with higher numbers of subjects. However, we must emphasise that GH therapy has been absolutely contraindicated for aesthetic or antiageing reasons until now.

## Figures and Tables

**Table 1:** Baseline characteristics range (placebo and GH group).

	Placebo (*n* = 6)		GH (*n* = 7)	
	Medium ± SD**	Range	Medium ± SD	Range
Age (years)	58 ± 4	51–63	56 ± 4	50–62
Weight (Kg)	81 ± 15	62–93.8	80 ± 13	62.7–104.7
BMI (kg/m^2^)*	26.0 ± 3.0	22.2–29	28.0 ± 6.0	20.7–38.5
IGF-I (ng/mL) (normal range: 78–258 ng/mL)	165 ± 35.36	120–228	216.7 ± 34.63	157–262
GH peak (*μ*g/L)	16.35 ± 9.53	6.34–37.8	16.1 ± 8.05	3.7–27.7

*BMI: body mass index; **SD: standard deviation.

**Table 2:** Evaluation before and after 6 months of treatment (placebo and GH).

	Placebo (*n* = 6)			GH (*n* = 7)		
	Baseline (medium ± SD*)	After 6 months (medium ± SD)	Δ	Baseline (medium ± SD)	After 6 months (medium ± SD)	Δ
Weight (kg)	81 ± 15	83 ± 16	1.3	80 ± 13	80 ± 15	0.4
BMI (kg/m^2^)	26 ± 3	26 ± 3	0.4	28 ± 6	28 ± 6	0.2
Waist (cm)	93 ± 10	94 ± 11	1.0	94 ± 9	96 ± 13	1.9
Σ folds** (mm)	55 ± 18	59 ± 21	4.2	64 ± 18	67 ± 23	3.4
Supine (10 MR)	36 ± 5	36 ± 4	0.0	31 ± 7	32 ± 8	1.3
Leg press (10 MR)	73 ± 12	77 ± 13	3.3	70 ± 14	84 ± 23^#^	**14.4** ^ ##^

*SD: standard deviation. **Sum of the cutaneous folds (triceps, biceps, subscapular, and iliac folds).

^#^Statistically significant difference in relation to pretest (ANOVA + Fisher LSD); *P* = 0.034. ^##^Statistically significant difference for Δ value (Student's *t*-test); *P* < 0.05.
